# A Herbal Composition of Semen Hoveniae, Radix Puerariae, and Fructus Schisandrae Shows Potent Protective Effects on Acute Alcoholic Intoxication in Rodent Models

**DOI:** 10.1155/2012/638197

**Published:** 2012-10-15

**Authors:** Jie Xiong, Yu Guo, Lu-yi Li, Hang Hu, Xin-lan Qu, Xi-zhen Sun, Sheng-hua Liu, Hui Wang

**Affiliations:** ^1^Department of Pharmacology, School of Basic Medical Science, Wuhan University, Wuhan 430071, China; ^2^Research and Development Center, Jing Brand Co., Ltd., Huangshi 435100, China; ^3^Research Center of Food and Drug Evaluation, Wuhan University, Wuhan 430071, China

## Abstract

This study is designed to evaluate the effects of a herbal composition of Semen Hoveniae, Radix Puerariae and Fructus Schisandrae (SRF) against acute alcoholic intoxication. The animals were treated with SRF extract (SRFE) for 14 days, and ethanol was conducted subsequent to the final treatment. The effects of SRFE on righting reflex, inebriety rates, kinetic parameters of blood ethanol and acetaldehyde were determined. In addition; levels of alcohol dehydrogenase (ADH) and aldehyde dehydrogenase (ALDH), the activities of cytochrome P450 2E1 (CYP2E1), selected antioxidative enzymes, and the contents of malonaldehyde (MDA) were measured. SRFE-pretreated rodents exhibited lower rates of intoxication, longer times to loss of righting reflex, and shortened times to recovery of righting reflex than in controls. The peak concentrations and area under the time-concentration curves were lower in the pretreated animals than in controls, which corresponded to higher levels of ADH and ALDH in both gastrointestines and livers of the SRFE-treated animals. The activities of CYP2E1 were lower in SRFE-pretreated animals, which also exhibited higher activities of some antioxidant enzymes and lower hepatic MDA levels. These findings suggest that the anti-inebriation effects of SRFE may involve inhibition of ethanol absorption, promotion of ethanol metabolism, and enhancing hepatic anti-oxidative functions.

## 1. Introduction

Alcohol intoxication has long been a prevalent phenomenon worldwide and is one that has led to severe problems, physically, economically, and socially [1–3]. Acute alcoholic intoxications caused by a single episode of excessive drinking may give rise to acidosis, potential heart failure, and autonomic and cerebral dysfunction, which ultimately leads to respiratory depression [4]. 

 In wake of the increasing alcohol consumption, people's demands for favorable medications against alcoholic intoxication have thereupon become strengthened in the past decades. In some western countries, a great number of synthetic drugs have been developed, nevertheless, serious existing side effects, such as addiction and behavior disorder, significantly limit their clinical usage [5–9]. However, Asian countries, particularly China, pay more attention to the application of natural medicines. Traditional Chinese medicines have been used safely and effectively to treat alcoholic intoxication for over two millennia due to the special features and satisfying efficacy. Natural medicines can be used exclusively or in combination, as each single herb or complex prescription contains a great variety of effective components, which presents a wide range of therapeutic spectra to various targets, thus gradually recovers the physical condition. Nowadays, several classic traditional single formulas, such as Semen Hoveniae, Radix Puerariae, Flos Puerariae, Fructus Schisandrae, Radix Glycyrrhizae, and Radix Salviae miltiorrhizae [10–14], in addition to complex formulas like “Hai-Wang-Jin-Zun,” “Ge-Hua-Jie-Cheng-Tang,” “Zhi-Ge-Yin,” “Xiao-Chai-Hu-Tang,” “Shi-Gao-Tang,” and “Wu-Lin-San” are still being used for treating alcohol intoxication [15–17].

The liver is one of the major target organs of ethanol actions. Alcohol abuse can lead to alterations in hepatocyte structure and functions. Several mechanisms contribute to alcohol-induced liver injury, but oxidative stress resulting from the toxic effects of reactive oxygen species (ROS) appears to be a primary pathway [18, 19]. In general, hepatocytes have antioxidant enzymes, such as superoxide dismutase (SOD), catalase (CAT), glutathione peroxidase (GPx), and glutathione S-transferase (GST), to attenuate injury caused by ROS and oxidative processes. Alcohol causes oxidative stress by both promoting the production of ROS and suppressing the activities of antioxidant enzymes, medications with opposite activities are highly anticipated. Since free radical generation by the ethanol-induced cytochrome P450 2E1 (CYP2E1) plays a key role in the oxidative stress, inhibitors of this enzyme also have great promise [19]. 

On the basis of the literature on herbal compounds that have inhibitory effects on acute alcoholic intoxication in the preliminary stage, we have created a formula comprising of Semen Hoveniae, Radix Puerariae, and Fructus Schisandrae (SRF). In a previous study, we optimized the combination and extraction of the three herbs, then evaluated the safety of this preparation by means of a maximum tolerable dose test (data not shown). In this way, a series of experiments were designed to test the anti-inebriation effects of SRF extract (SRFE) and to explore in rodent models the underlying mechanisms against cute alcoholic intoxication, from the perspective of inhibition of ethanol absorption, promotion of ethanol metabolism, and improved hepatic antioxidative functions. Well-known as the specific medicine used exclusively for acute alcoholic intoxication [20], Semen Hoveniae (SH) is considered as the primary drug in SRF. Therefore, we also tested the effects of SRF, by comparing the protective effects of SRF and SH.

## 2. Materials and Methods

### 2.1. Drugs and Reagents

 As a positive drug for alcoholic intoxication, Hai-Wang-Jin-Zun tablets (HWJZ) were obtained from Shenzhen Neptunus Group Co., Ltd. (Shenzhen, China). High-performance liquid chromatography (HPLC) grade acetonitrile was purchased from Thermo Fisher Scientific, Inc. (PA, USA). Standard substances (quercetin, rutin, puerarin, kaempferol, *γ*-schizandrin, and schisandrin, purities > 95%) were purchased from the National Institute for Food and Drug Control (Beijing, China). Rat aldehyde dehydrogenase (ALDH) ELISA kits were purchased from Research and Diagnostics Systems, Inc. (MN, USA). Alcohol dehydrogenase (ADH), SOD, CAT, GPx, GST, and malondialdehyde (MDA) kits were obtained from Nanjing Jiancheng Bioengineering Institute (Nanjing, China). All other chemicals were analytical reagent and purchased from National Chemical Co. (Shanghai, China).

### 2.2. Plants

 The medicinal plants used in SRF include the seed of *Hoveniadulcis*  Thunb., the root of *Pueraria lobata* (Willd.) Ohwi and the fruit of *Schisandra chinensis* (Turcz.) Baill. They were purchased from Zhongnan Hospital of Wuhan University (Wuhan, China), and authenticated by pharmaceutist Zhuan Zhang (Department of Pharmacy, Zhongnan Hospital of Wuhan University). All voucher specimens were deposited in our laboratory for future reference (no. 100901, 100902, 100903).

### 2.3. Preparation of Extracts

The SRFE and SH extract (SHE) were prepared according to the procedure below: for SRFE, Semen Hoveniae 40 g, Radix Puerariae 30 g, and Fructus Schisandrae 15 g were immersed in 680 mL of 70% ethanol and reflux-extracted for 1.5 hours. After filtering, the residue was extracted for 1 hour and filtered again. The two extracts were combined and concentrated to dryness. The yield of SRFE was 7.0 g (8.2%, w/w), and the total contents of flavonoids was 46.3 mg/g SRFE. For SHE, 85 g of Semen Hoveniae were extracted by the same method, and the yield of SHE is 6.8 g (8.0%, w/w). 

**Figure 1:**
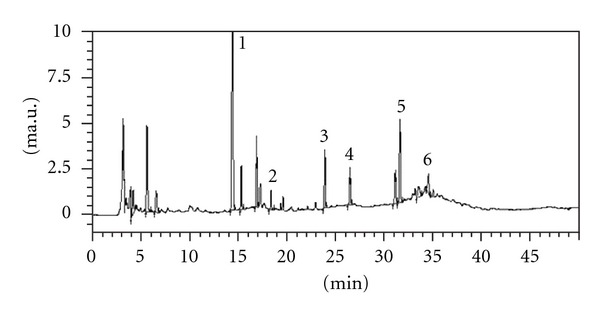
HPLC chromatogram of Semen Hoveniae, Radix Puerariae, and Fructus Schisandrae extract. 1: Puerarin; 2: Rutin; 3: Quercetin; 4: Kaempferol; 5: Schisandrin; 6: *γ*-Schizandrin. Each peak was identified by comparison with standard compounds.

 The HPLC analysis of SRFE was carried out by a Shimadzu LC-20AT system (Tokyo, Japan). The separation was performed on a C_18_ column (250 mm × 4.6 mm, 5.0 *μ*m; Dikma, Beijing, China). The sample injection volume was 5 *μ*L. The flow rate was kept at 1 mL/min. The mobile phases consisted of 0.2% phosphoric acid (A) and acetonitrile (B). The system was run with the following gradient program: 90% A for 5 min; from 90% A to 70% A for next 10 min; 70% A for 15 min; from 70% A to 90% A for 10 min; and then kept at 90% A for 10 min. Absorbance was measured at 250 nm, and the major compounds and the respective concentrations in SRFE were puerarin (19.217 mg/g SRFE), rutin (0.189 mg/g SRFE), quercetin (19.082 mg/g SRFE), kaempferol (0.310 mg/g SRFE), schisandrin (3.985 mg/g SRFE), and *γ*-schizandrin (0.017 mg/g SRFE) (Figure 1). 

### 2.4. Animals

 Specefic pathogen-free grade male Kunming mice weighing 20 ± 2 g and Wistar rats weighing 200 ± 20 g were obtained from Hubei Academy of Preventive Medicine (Wuhan, China) and acclimatized for one week before the experiments. Animals were fed under controlled conditions (24 ± 2°C, 12 : 12-h dark-light cycle) with free access to regular chow and water. All animal experiment procedures were approved by and performed in accordance with the Guidelines for the Care and Use of Laboratory Animals of the Chinese Animal Welfare Committee.

### 2.5. Mouse Experiment for Pharmacodynamic Study

 Mouse experiments were designed to determine the effects of SRFE on acute alcoholic intoxication. For SRFE groups, mice were intragastrically treated with low (0.06 g/kg), medium (0.12 g/kg), and high (0.24 g/kg) doses of SRFE, respectively. Meanwhile, control and model groups received equal volumes of distilled water. SRFE control animals received the high dose of SRFE. For the HWJZ group, mice received 0.40 g/kg HWJZ. After 14 consecutive days of drug administration, 7.70 g/kg ethanol was administered orally one hour subsequent to the final treatment with SRFE groups as well as model and HWJZ groups. The times to loss of righting reflex and the times to recovery of righting reflex were recorded [16]. For those animals that did not become inebriated, the time of loss and recovery of righting reflex are set to 500 and 0 min, respectively. The rate of intoxication (%) was (the number of inebriated mice/the number of inebriated and noninebriated mice) × 100. Mice were euthanized 12 hours later under isoflurane anesthesia, and the livers were obtained for the assays of antioxidative enzymes.

### 2.6. Rat Experiment for Pharmacokinetic Study

 Rat experiments were designed to study the effects of SRFE on pharmacokinetics of ethanol. Drug administration protocols were identical with mouse experiment, and the dosages used in this part were calculated based on body surface area quotiety between mouse and rat [21]. The doses of SRFE were 0.04 (low dose), 0.08 (medium dose), and 0.16 (high-dose) g/kg, and the doses of HWJZ and ethanol were 0.25 and 4.50 g/kg. In this study, blood was obtained from the *vena caudalis* under light isoflurane anesthesia 0, 0.5, 1, 2, and 6 hours after ethanol treatment, and the serum was subsequently separated for detecting the concentrations of ethanol and its main metabolite, acetaldehyde. Kinetic parameters of blood ethanol and acetaldehyde, including time to peak concentration (*T*_max⁡_), peak concentration (*C*_max⁡_), and area under the curve (AUC) were recorded and analyzed. Rats were euthanized 12 hours later under isoflurane anesthesia, the stomachs, small intestinal mucosa, and livers were obtained for the assays of ethanol metabolic enzymes. 

### 2.7. Blood Ethanol and Acetaldehyde Concentrations

 Quantitation of ethanol and acetaldehyde in rat serum were carried out by the headspace gas chromatography mass spectrometry in selected ion monitoring mode. Agilent (CA, USA) 7890A GC System and 5975C inter XL MSD were used, and the separation was performed on a capillary column (CP-WAX 57CB, Agilent). Analytical grade ethanol and acetaldehyde were used as standard to plot standard curves with the concentration ranges of 1–110 *μ*mol/L (*n* = 10) and 4.5–675 *μ*mol/L (*n* = 10), respectively. To 20-mL HS glass vials were added 100 *μ*L serum, 1.2 g NaCl, and 10 *μ*L internal standard solution (0.1% amylene hydrate), and the samples were incubated at 80°C for 30 min. The sample injection volume was 1 mL. Constant column flow of carrier gas was 1.22 mL/min, and column temperature was 50°C for 12 min. 

### 2.8. Other Indices Assay

 According to the manufacturer's protocols, rat ALDH ELISA kits were used to determine the contents of ALDH in livers, stomachs, and mucous membrane of small intestines of the animals. The contents of MDA and the activities of SOD, CAT, GPx, and GST in liver homogenates, as well as ADH activities and protein contents in tissue homogenates were determined by of the respective commercial kits. Aniline hydroxylase activities were detected as reflective of CYP2E1, as described previously [22].

### 2.9. Statistics

 Data on the loss and recovery of righting reflex were compared using the Wilcoxon signed-rank test, and inebriety rates were analyzed by means of Fisher's exact test. Results were presented as means ± S.E.M. and were analyzed by one-way ANOVA, followed by Dunnett's  *t-*test. Statistical significance was determined at *P* < 0.05. 

## 3. Results

### 3.1. Righting Reflex and Rate of Intoxication in Mice (Table 1) 

SRFE pretreated animals exhibited longer times to ethanol-induced loss of righting reflex and shorter times to recovery of righting reflex, and these effects of SRFE were dose-dependent. The inebriety rates were lower (*P* < 0.05) in the high-dose (0.24 g/kg) SRFE animals than in the vehicle-treated models. The positive control drug HWJZ (0.4 g/kg) showed effects that were similar to the effects observed in animals treated with the medium dose (0.12 g/kg) of SRFE. Thus, SRFE increased ethanol tolerance in mice as well as did HWJZ. 

### 3.2. Kinetic Parameters of Serum Ethanol and Acetaldehyde in Rats (Table 2) 

*C*_max⁡_ and AUC of serum ethanol were lower (*P* < 0.05, *P* < 0.01) in medium (0.08 g/kg) and high (0.16 g/kg) doses-treated SRFE animals than in models, but *T*_max⁡_ values were not different. The *C*_max⁡_ and AUC of serum acetaldehyde were lower in animals treated with the high dose of SRFE (*P* < 0.01), while *T*_max⁡_ values were remarkably shorter in the animals treated with the medium dose of SRFE (*P* < 0.05) than in models. In rats pretreated with HWJZ (4.50 g/kg), only the *T*_max⁡_ of serum ethanol and acetaldehyde were reduced (*P* < 0.05). Thus, SRFE can decrease the absorbed level of ethanol, promote acetaldehyde generation, and reduce the contents of acetaldehyde in blood, while HWJZ can accelerate the absorption of ethanol and the generation of acetaldehyde. 

### 3.3. ADH and ALDH Levels in Rats (Table 3) 

 Compared with the rats in the control group, the levels of ADH, and ALDH in the stomach and small intestine were invariant in the rats of SRFE control group except for the stomach ADH activities (*P* < 0.05), while these two enzymes in the liver were increased (*P* < 0.01). The ADH activities and the ALDH contents for the rats in the model group were found to decrease in the stomach and small intestine but increased in the liver, especially for the ALDH content (*P* < 0.01, *P* < 0.05). Compared to the model group, ADH and ALDH levels in stomach, small intestine, and liver were dose-dependently enhanced by pretreating with SRFE, the changes were significant in stomach and small intestine for high-dose (0.16 g/kg) treated rats (*P* < 0.01, *P* < 0.05). In the HWJZ group, ADH activities and ALDH contents were only raised in the liver (*P* < 0.01, *P* < 0.05). These results implied that ADH and ALDH levels in the gastrointestinal tract and liver could be elevated by SRFE in inebriated rats.

### 3.4. Hepatic CYP2E1 Activity in Rats (Figure 2)

 The activities of hepatic CYP2E1 were increased (*P* < 0.01) in ethanol treated rats compared to the controls, and nearly invariant in the rats of the SRFE control group. The elevation of hepatic CYP2E1 activity induced by ethanol was dose-dependently decreased by pretreating with SRFE. In rats in the high-dose (0.16 g/kg) treated group, the changes were significant (*P* < 0.01), and the levels of CYP2E1 were approximately equal to those in the control group. HWJZ showed effects that were similar to the effects observed in animals treated with the medium dose (0.08 g/kg) of SRFE (*P* < 0.05). These results implied that SRFE as well as HWJZ could inhibit CYP2E1 activity in the liver of inebriated rats. 

### 3.5. Hepatic Antioxidative Activities in Mice (Table 4)

 Compared to those of the control group, the mice in the SRFE control group showed little change in the levels of hepatic antioxidative enzymes and MDA, while the activities of hepatic SOD, CAT, GPx, and GST all decreased in mice of the model group (*P* < 0.05, *P* < 0.01). These inhibitory effects of ethanol on SOD, CAT, and GST were reversed by all doses of SRFE as well as HWJZ (*P* < 0.05, *P* < 0.01), while for GPx, the activities were raised only in high (0.24 g/kg) and low (0.06 g/kg) doses of SRFE. MDA contents increased in the model group (*P* < 0.05), and this elevation was reduced by medium (0.12 g/kg) and high doses of SRFE (*P* < 0.01, *P* < 0.05). The levels of SOD, CAT, and MDA were approximately brought to the control group level by SRFE and HWJZ. Thus, hepatic antioxidative function was enhanced, and lipid peroxidation product was reduced by pretreatment with SRFE and HWJZ, especially by SRFE. 

### 3.6. Comparisons of SRFE and SHE (Table 5). 

 Compared with the model group, the times of loss of righting reflex were extended (*P* < 0.01), and the times of recovery of righting reflex were shortened (*P* < 0.01), meanwhile, serum ethanol concentrations and hepatic MDA contents were reduced in SRFE (0.24 g/kg) group (*P* < 0.01, *P* < 0.05). SHE (0.24 g/kg) showed effects that were less pronounced than the effects observed in animals treated with SRFE, indicating that SRFE showed a better protective effect than SHE against acute alcoholic intoxication. 

## 4. Discussion

 As the typical natural medicines, Semen Hoveniae, Radix Puerariae, and Fructus Schisandrae are used in many classic traditional Chinese single and complex formulas on account of their antialcoholic activities and protective effects against hepatic injury [16, 23]. Based on previous clinical experiences and animal tests, we combined the three herbs to form SRF for the first time. According to Traditional Chinese Medicine theory, there existed no incompatibility among the three herbs when compounded to a complex formula, which possessed comprehensive pharmacological effects, such as clearing heat, accelerating diuresis, dissolving stasis, eliminating toxins, and regulating the function of liver and stomach. Recent research suggests that Semen Hoveniae is able to increase the levels of hepatic ADH and ALDH in alcohol-induced acute liver injury mice [10, 24], while Radix Puerariae can normalize the activities of hepatic antioxidative enzymes in rodent alcoholic intoxication models [24, 25], and Fructus Schisandrae displays potent hepatoprotective effect in CCl_4_-induced rat hepatic injury [23, 26]. In this study, it was shown that SRFE extended the time to loss of the righting reflex and shortened the time of recovery of the righting reflex, lowered the inebriety rate, and reduced the hepatic MDA generation.

 Ethanol is almost completely absorbed from the gastrointestinal tract and eliminated mainly *via* hepatic metabolism [27]. The hepatocytes contain three main pathways for ethanol metabolism: (1) the ADH pathway of the cytosol, (2) the microsomal ethanol oxidizing system (MEOS) located in the endoplasmic reticulum, and (3) catalase located in the peroxisomes [10]. A small dose of ethanol in liver is oxidized to acetate catalyzed by ADH and ALDH, while a chronic or a high dose elevates the hepatic activity of CYP2E1, and accelerates ROS and toxic metabolite generation resulting in damaging liver [28]. Although each of the pathways produces specific metabolites and even toxins, all result in the production of acetaldehyde, a highly toxic metabolite that can only be catalyzed by ALDH to generate acetate, and finally be oxidized to CO_2_ and H_2_O. It has been reported that acetaldehyde accumulation has serious hepatotoxic effects, such as covalent binding to proteins, microtubular and mitochondrial impairment, lipid peroxidation, pyridoxine depletion, increased collagen synthesis, inhibition of DNA repair, and stimulation of immunologic reactivity, causing considerable harm to the liver [19]. In addition to hepatocytes, ADH and ALDH are expressed in the stomach and small intestine as well, decomposing ethanol prior its absorption [29]. Therefore, oxidases in both gastrointestine and liver play a crucial role in the process of alcohol absorption and metabolism. In this study, it has been demonstrated that SRFE has antialcoholic intoxication activity by (1) enhancing the gastrointestinal first-pass effect and reducing ethanol absorption *via *elevating the gastrointestinal levels of ADH and ALDH, (2) increasing hepatic ADH and ALDH levels to enhance the ADH-dependent pathway for ethanol metabolism, and (3) reducing hepatic CYP2E1 activity to inhibit the MEOS pathway and the generation of ROS and other toxic metabolites.

 Several mechanisms contribute to alcohol-induced liver injury, but the generation of ROS (e.g., O_2_^•−^, H_2_O_2_) appears to be a primary pathway [18]. Excess ROS are toxic to hepatocytes since they can react with most cellular macromolecules and cause liver damage, ultimately, a harmful condition known as oxidative stress occurs. Superoxide (O_2_^•−^) has been proved to play a central role in alcohol-induced liver injury. Not only is O_2_^•−^ readily produced by numerous processes *in vivo*, but also many other oxidants found *in vivo* are derived from O_2_^•−^ [30]. In general, cells have intrinsic protective mechanisms to attenuate injury caused by ROS and the oxidative process. Some enzymes like SOD (which converts O_2_^•−^ to H_2_O_2_), CAT and GPx (which remove H_2_O_2_), and GST (which removes active intermediate products that promote macromolecules damage) are involved in these mechanisms mentioned above. Alcohol causes oxidative stress by both promoting the production of oxidative species and suppressing antioxidant enzymes [18]. In this study, SRFE has exhibited the antioxidant activity and hepatoprotective effect by inducing the activities of hepatic antioxidant enzymes (e.g., SOD, CAT, GPx, and GST) and by reducing the generation of lipid peroxidation product (e.g., MDA), which significantly reversed the oxidative stress induced by alcohol.

 HWJZ which boasts a very high market share in China is a natural drug with antialcoholic intoxication and hepatoprotective effects. In this study, it has been found that HWJZ has some positive effects, for example, shortening the sleep duration of inebriated mice. The realization of the mechanisms may be attributed to accelerating the absorption of ethanol and the generation of acetaldehyde, inhibiting hepatic CYP2E1 activity, and enhancing the activities of hepatic antioxidative enzymes. In contrast, SRFE has showed more comprehensive effects, mainly on: (1) postponing the inebriated time, shortening the sleep duration, and reducing the degree of inebriation. As the result of increased levels of ADH and ALDH in the stomach, small intestine, and liver, these effects might be relevant to inhibiting the absorption of ethanol as well as promoting ethanolic metabolism; (2) exhibiting a prominent effect against liver oxidative damage which might be attributed to inhibiting hepatic CYP2E1 activity, enhancing the activities of hepatic antioxidative enzymes, and reducing the generation of lipid peroxide. In addition, as a traditional plant medicine used exclusively for acute alcoholic intoxication [20], SH is considered as the primary drug in SRF. It has been found that compared to SHE, SRFE possesses superior anti-intoxication effect and protection against hepatic injury in an acute *in vivo* intoxication model.

## 5. Conclusions

 This study has demonstrated that SRFE has a favorable protection against acute ethanol intoxication in rodent models. The mechanisms that contribute to this effect might be attributed to the inhibition of ethanol absorption, promotion of ethanol metabolism, and improved hepatic antioxidative function.

## Figures and Tables

**Figure 2:**
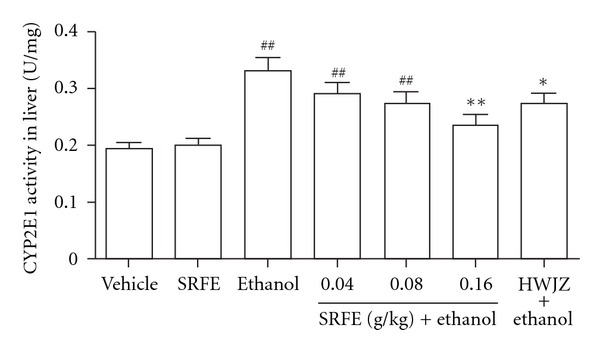
Effect of SRFE on hepatic CYP2E1 activity in acutely inebriated rats. CYP2E1: cytochrome P450 2E1; SRFE: Semen Hoveniae, Radix Puerariae, and Fructus Schisandrae extract; HWJZ: Hai-Wang-Jin-Zun tablet. Means ± S.E.M., *n* = 9. ^##^ *P* < 0.01 versus control; *P < 0.05, **P < 0.01 versus model.

**Table 1:** Effects of SRFE on righting reflex and rate of intoxication in acutely inebriated mice.

Groups	Dose	Ethanol	Loss of the righting reflex	Recovery of the righting reflex	Rate of intoxication
	(g/kg)	(g/kg)	(mean rank)	(mean rank)	(%)
Model	—	7.70	28.14	51.64	78
SRFE	0.06	7.70	38.93	38.53	53
	0.12	7.70	43.77^∗^	36.13	47
	0.24	7.70	45.31^∗^	31.31^∗∗^	38^∗^
HWJZ	0.40	7.70	40.85	34.81^∗^	46

SRFE: Semen Hoveniae, Radix Puerariae, and Fructus Schisandrae extract; HWJZ: Hai-Wang-Jin-Zun tablet. *n* = 12. *P < 0.05, **P < 0.01 versus model.

**Table 2:** Effects of SRFE on serum ethanol and acetaldehyde concentrations in acutely inebriated rats.

Groups	Doses	Ethanol	Ethanol				Acetaldehyde		
	(g/kg)	(g/kg)	*T*_max⁡_ (h)	*C*_max⁡_ (g/L)	AUC (g*·*h/L)		*T*_max⁡_ (h)	*C*_max⁡_ (mg/L)	AUC (mg*·*h/L)
Model	—	4.50	2.94 ± 0.78	2.40 ± 0.31	8.12 ± 1.14		3.11 ± 0.73	73 ± 6	316 ± 38
SRFE	0.04	4.50	2.06 ± 0.76	3.55 ± 0.39^∗^	12.89 ± 1.57^∗^		2.61 ± 0.67	68 ± 6	332 ± 36
	0.08	4.50	2.94 ± 0.78	1.52 ± 0.15^∗^	5.38 ± 0.49^∗^		1.28 ± 0.25^∗^	53 ± 4^∗^	226 ± 17
	0.16	4.50	2.05 ± 0.53	1.35 ± 0.18^∗∗^	4.81 ± 0.91^∗^		1.50 ± 0.58	43 ± 5^∗∗^	159 ± 16^∗∗^
HWJZ	0.25	4.50	0.83 ± 0.17^∗^	2.37 ± 0.35	8.08 ± 1.14		1.11 ± 0.23^∗^	74 ± 10	265 ± 33

SRFE: Semen Hoveniae, Radix Puerariae, and Fructus Schisandrae extract; HWJZ: Hai-Wang-Jin-Zun tablet; *T*_max⁡_: time to peak concentration; *C*_max⁡_: peak concentration; AUC: area under the curve. Means ± S.E.M., *n* = 9. *P < 0.05, **P < 0.01 versus model.

**Table 3:** Effects of SRFE on ADH and ALDH levels in acutely inebriated rats.

Groups	Doses (g/kg)	Ethanol (g/kg)	Stomach		Small intestine		Liver	
			ADH (U/g prot)	ALDH (*μ*mol/L)	ADH (U/g prot)	ALDH (*μ*mol/L)	ADH (U/g prot)	ALDH (*μ*mol/L)
Control	—	0.00	0.95 ± 0.03	0.39 ± 0.01	2.75 ± 0.20	1.65 ± 0.06	7.56 ± 0.33	6.31 ± 0.08
SRFE control	0.16	0.00	1.10 ± 0.05^#^	0.42 ± 0.01	3.12 ± 0.14	1.73 ± 0.11	9.56 ± 0.34^##^	8.10 ± 0.10^##^
Model	—	4.50	0.85 ± 0.07	0.36 ± 0.02^#^	2.49 ± 0.26	0.46 ± 0.08^##^	8.87 ± 0.57	7.92 ± 0.33^##^
SRFE	0.04	4.50	0.97 ± 0.05	0.38 ± 0.01	2.75 ± 0.27	0.45 ± 0.05	8.92 ± 0.45	7.91 ± 0.21
	0.08	4.50	0.97 ± 0.05	0.41± 0.02^∗^	2.91 ± 0.22	0.57 ± 0.10	9.29 ± 0.53	8.11 ± 0.28
	0.16	4.50	1.07± 0.05^∗^	0.41± 0.01^∗^	3.27 ± 0.22^∗^	1.19 ± 0.14^∗∗^	9.77 ± 0.35	8.27 ± 0.26
HWJZ	0.25	4.50	0.97 ± 0.06	0.36 ± 0.01	2.81 ± 0.22	0.80 ± 0.14	10.47 ± 0.50^∗^	9.95 ± 0.24^∗∗^

SRFE: Semen Hoveniae, Radix Puerariae, and Fructus Schisandrae extract; HWJZ: Hai-Wang-Jin-Zun tablet; ADH: ethanol dehydrogenase; ALDH: acetaldehyde dehydrogenase. Means ± S.E.M., *n* = 9. ^#^ *P* < 0.05, ^##^ *P* < 0.01 versus control; *P < 0.05, **P < 0.01 versus model.

**Table 4:** Effects of SRFE on activities of antioxidative enzymes and content of MDA in the liver of acutely inebriated mice.

Groups	Doses	Ethanol	SOD	CAT	GPx	GST	MDA
	(g/kg)	(g/kg)	(U/mg prot)	(U/mg prot)	(U/mg prot)	(U/mg prot)	(*μ*mol/g prot)
Control	—	0.00	245 ± 7	85 ± 2	386 ± 17	55 ± 3	1.20 ± 0.03
SRFE control	0.24	0.00	245 ± 5	84 ± 3	424 ± 14	57 ± 2	1.15 ± 0.04
Model	—	7.70	203 ± 6^##^	74 ± 3^#^	318 ± 13 ^##^	37 ± 2^##^	1.33 ± 0.03^#^
SRFE	0.06	7.70	226 ± 6^∗^	89 ± 2^∗∗^	356 ± 11^∗^	49 ± 2^∗∗, #^	1.23 ± 0.04
	0.12	7.70	225 ± 6^∗^	88 ± 3 ^∗∗^	325 ± 17^#^	46 ± 2^∗∗, #^	1.16 ± 0.06^∗^
	0.24	7.70	230 ± 5^∗∗^	91 ± 3 ^∗∗^	376 ± 13 ^∗∗^	46 ± 3^∗∗, #^	1.14 ± 0.04^∗∗^
HWJZ	0.40	7.70	228 ± 7^∗^	88 ± 4^∗∗^	315 ± 21^#^	44 ± 3^∗, #^	1.24 ± 0.05

SRFE: Semen Hoveniae, Radix Puerariae, and Fructus Schisandrae extract; HWJZ: Hai-Wang-Jin-Zun tablet; SOD: superoxide dismutase; CAT: catalase; GPx: glutathione peroxidase; GST: glutathione S-transferase; MDA: malondialdehyde. Means ± S.E.M., *n* = 12. ^#^ *P* < 0.05, ^##^ *P* < 0.01 versus control. *P < 0.05, **P < 0.01 versus model.

**Table 5:** Comparison of the anti-inebriation effects and changes in hepatic antioxidative capacity induced by SRFE and SHE in acutely inebriated mice.

Groups	Doses	Ethanol	Loss of the righting reflex	Recovery of the righting reflex	Serum ethanol	Hepatic MDA
	(g/kg)	(g/kg)	(mean rank)	(mean rank)	(g/L)	(*μ*mol/g prot)
Model	—	7.70	19.22	31.75	3.77 ± 0.22	1.37 ± 0.05
SRFE	0.24	7.70	29.75^∗^	18.91^∗∗^	2.05 ± 0.5^ ∗∗^	1.21 ± 0.03^∗^
SHE	0.24	7.70	26.87	23.40	3.35 ± 0.11	1.31 ± 0.04

SRFE: Semen Hoveniae, Radix Puerariae, and Fructus Schisandrae extract; SHE: Semen Hoveniae extract; MDA: malondialdehyde. Means ± S.E.M., *n* = 12. *P < 0.05, **P < 0.01, versus model.

## Abbreviations

ADH: Alcohol dehydrogenase
ALDH: Aldehyde dehydrogenase
AUC: Area under the curve
CAT: Catalase
*C*_max⁡_: Peak concentration
CYP2E1: Cytochrome P450 2E1
GPx: Glutathione peroxidase
GST: Glutathione S-transferase
HPLC: High-performance liquid chromatography
HWJZ: Hai-Wang-Jin-Zun tablet
MDA: Malonaldehyde
MEOS: Microsomal ethanol oxidizing system
ROS: Reactive oxygen species
SH: Semen Hoveniae
SHE: Semen Hoveniae extract
SOD: Superoxide dismutase
SRF: Semen Hoveniae, Radix Puerariae and Fructus Schisandrae
SRFE: Semen Hoveniae, Radix Puerariae and Fructus Schisandrae extract
*T*_max⁡_: Time to peak concentration.
